# Use of immunosuppressive medications and prevalence of disability among patients with pediatric systemic lupus erythematosus: initial analysis of the CARRAnet registry

**DOI:** 10.1186/1546-0096-10-S1-A28

**Published:** 2012-07-13

**Authors:** Aimee Hersh, Mary Beth F Son, Hermine Brunner, Barbara Anne Eberhard, Emily Von Scheven

**Affiliations:** 1 CARRA, Stanford, CA, USA; 2 Children's Hospital Boston, Boston, MA, USA; 3 Cincinnati Children's Hospital Medical Center, Cincinnati, OH, USA; 4 Cohen Children's Medical Center, New Hyde Park, NY, USA; 5 University of CA San Francisco, San Francisco, CA, USA; 6 University of Utah, Salt Lake City, UT, USA

## Purpose

To describe the demographics, disease features and treatment of participants with pediatric systemic lupus erythematosus (pSLE) enrolled to date in the CARRAnet registry.

## Methods

Subjects were included in the CARRAnet registry, a national pediatric rheumatology database, if diagnosed with pSLE at ≤16 years of age and ≤ age 21 at enrollment. Demographics, disease features and prior and current treatments were collected, as well as the SLE disease activity index (SLEDAI), Health Related Quality of Life (HRQOL), ACR Functional Class (current and worst ever), Childhood Health Assessment Questionnaire (CHAQ), and Physician Global Assessment of disease activity (PGA). Associations between parameters were assessed using Spearman’s coefficient, and linear regression was used to determine predictors of intravenous (IV) cyclophosphamide (CYC) administration.

## Results

Data from 123 participants enrolled at 20 sites were analyzed; 2 sites contributed 43 participants (35%). Mean age at onset of symptoms was 13 ± 3yrs (range 3.3-19.9 yrs), and mean age at enrollment was 16.3 yrs ± 2.9 (range 7.1-21.4 yrs). 42 subjects (34%) were diagnosed at ≤12 yrs of age. The cohort was 34.1% white, 40.7% black, 7.3% Asian, 3.3% American Indian, and 14.6% of participants were classified as ‘other’. 22 participants (17.8%) were Hispanic. In the prior 6 months, 3.3% (n=4) had pulmonary involvement, 4.0% (n=5) had myocarditis/pericarditis, and 15.4% (n=19) developed at least 1 of the following: lupus headache, cranial nerve disorder, organic brain syndrome, psychosis and/or seizure. Since diagnosis, 57 participants (46.3%) underwent renal biopsy, avascular necrosis developed in 10 (8.1%), and thrombosis in 9 (7.3%). A summary of medications prescribed are listed in Table 1.

**Table 1:** Prescribed treatments in the CARRAnet registry

Medications	Ever prescribed N (%)	Currently prescribed N (%)
Prednisone	119 (97)	94 (79)
Methylprednisolone (IV)	66 (54)	4 (3)
Hydroxychlorquine	116 (94)	105 (85)
Mycophenolate mofetil	72 (59)	61 (50)
Cyclophosphamide (IV)	60 (49)	10 (8)
Azathioprine	27 (22)	10 (8)
Methotrexate (oral or injectable)	32 (26)	15 (12)
Rituximab	26 (21)	8 (7)

CYC use was not associated with gender, age at onset, CHAQ score, or ACR Functional Class; but was associated with renal biopsy (p<.001 [CI 4.14-30.6]). 56 participants (45.5%) were classified as ACR Functional Class III or IV at some point in their disease course, while14 (11.4%) were considered Class III or IV at the time of enrollment. 54 participants (43.9%) rated their HRQOL as “very good” or “excellent” but physical function was impaired with CHAQ>0 in 48% (n=59). The median score for PGA and SLEDAI was 2 (range 0-7) and 2 (range 0-38), respectively. There was a significant association between the CHAQ and SLEDAI scores (rho=0.19, p=0.03) or the PGA (rho=0.26, p=0.04).

## Conclusion

In the initial analysis of pSLE subjects in the CARRAnet registry, most participants required steroids for disease control and the majority were on additional immunosuppressants. Furthermore, disability occurred in nearly half the cohort, indicating improved treatment strategies are needed to treat pSLE.

## Disclosure

Aimee Hersh: None; Mary Beth F. Son: None; Hermine Brunner: None; Barbara Anne Eberhard: None; Emily Von Scheven: None; CARRAnet Investigators: None.

